# High Shedding Potential and Significant Individual Heterogeneity in Naturally-Infected Alpine ibex (*Capra ibex*) With *Brucella melitensis*

**DOI:** 10.3389/fmicb.2018.01065

**Published:** 2018-05-28

**Authors:** Sébastien Lambert, Emmanuelle Gilot-Fromont, Pauline Freycon, Anne Thébault, Yvette Game, Carole Toïgo, Elodie Petit, Marie-Noëlle Barthe, Gaël Reynaud, Maryne Jaÿ, Bruno Garin-Bastuji, Claire Ponsart, Jean Hars, Sophie Rossi

**Affiliations:** ^1^UMR Centre National de la Recherche Scientifique 5558 Biometry and Evolutionary Biology Laboratory, University of Lyon1, Villeurbanne, France; ^2^VetAgro Sup- Lyon Veterinary Campus, University of Lyon, Marcy l'Étoile, France; ^3^Risk Assessment Department, French Agency for Food, Environmental and Occupational Health and Safety (ANSES), Maisons-Alfort, France; ^4^Departmental Veterinary Laboratory of Savoie (LDAV 73), Chambéry, France; ^5^Mountain Wildlife Unit, French Hunting and Wildlife Agency (ONCFS), Gières, France; ^6^Mountain Wildlife Unit, French Hunting and Wildlife Agency (ONCFS), Sèvrier, France; ^7^EU/OIE/FAO & National Reference Laboratory for Animal Brucellosis, Animal Health Laboratory, French Agency for Food, Environmental and Occupational Health and Safety (ANSES)/Paris-Est University, Maisons-Alfort, France; ^8^Wildlife Diseases Unit, French Hunting and Wildlife Agency (ONCFS), Gières, France; ^9^Wildlife Diseases Unit, French Hunting and Wildlife Agency (ONCFS), Gap, France

**Keywords:** *Brucella melitensis*, Alpine ibex (*Capra ibex*), wildlife disease, serology, bacteriology, pathogenesis, transmission, epidemiology

## Abstract

Wildlife reservoirs of infectious diseases raise major management issues. In Europe, brucellosis has been eradicated in domestic ruminants from most countries and wild ruminants have not been considered important reservoirs so far. However, a high prevalence of *Brucella melitensis* infection has been recently identified in a French population of Alpine ibex (*Capra ibex*), after the emergence of brucellosis was confirmed in a dairy cattle farm and two human cases. This situation raised the need to identify the factors driving the persistence of *Brucella* infection at high prevalence levels in this ibex population. In the present paper, we studied the shedding pattern of *B. melitensis* in ibex from Bargy Massif, French Alps. Bacteriological examinations (1–15 tissues/samples per individual) were performed on 88 seropositive, supposedly infected and euthanized individuals. Among them, 51 (58%) showed at least one positive culture, including 45 ibex with at least one *Brucella* isolation from a urogenital sample or a lymph node in the pelvic area (active infection in organs in the pelvic area). Among these 45 ibex, 26 (30% of the total number of necropsied animals) showed at least one positive culture for a urogenital organ and were considered as being at risk of shedding the bacteria at the time of capture. We observed significant heterogeneity between sex-and-age classes: seropositive females were most at risk to excrete *Brucella* before the age of 5 years, possibly corresponding to abortion during the first pregnancy following infection such as reported in the domestic ruminants. The high shedding potential observed in young females may have contributed to the self-sustained maintenance of infection in this population, whereas males are supposed to play a role of transmission between spatial units through venereal transmission during mating. This heterogeneity in the shedding potential of seropositive individuals should be considered in the future to better evaluate management scenarios in this system as well as in others.

## Introduction

The transmission of infectious diseases results from a complex interplay between the pathogen, the host and the environment, which generates highly variable dynamics at all scales from individuals to populations or communities (Tompkins et al., 2011). In directly transmitted infectious diseases, heterogeneity of infectiousness has been neglected until some evidence of superspreading, i.e., extreme heterogeneity, where the 20% most infectious individuals may be responsible for more than 80% of cases, has been found for example for the Severe Acute Respiratory Syndrome in humans (SARS, Galvani and May, 2005) or for *Escherichia coli* O157 in cattle (Matthews et al., 2006). Generally speaking, heterogeneity increases the growth rate of outbreaks and the probability of stochastic extinction of the pathogen, and lowers the efficacy of control measures (Lloyd-Smith et al., 2005). Such heterogeneity may result from various factors, such as host age (Treanor et al., 2011), sex (Silk et al., 2018), immunity (Pathak et al., 2010), behavior (Drewe, 2010), or genetic background (Borriello et al., 2006).

Identifying the categories of individuals that are most responsible for disease transmission is a key toward targeting these individuals for efficient disease control (Matthews et al., 2006). Assessing the health status of individuals, which is often based on serological assays in wildlife, is not sufficient to attain this goal. Instead, it is necessary to assess within-individual pathogen distribution to infer a shedding pattern and individual heterogeneity in infectiousness (González-Barrio et al., 2015).

Here we deal with brucellosis, a major zoonosis that causes economic and public health issues worldwide. Infections with *Brucella abortus* or *Brucella melitensis* in ruminants mainly lead to late-term abortions and infertility, with substantial shedding of bacteria in the environment through genital fluids. Until recently, European wild ungulates were seen as dead-end hosts and had been thus considered as negligible hosts in Europe (Godfroid et al., 2013). In particular, previous cases of *B. melitensis* in European wild mountain ruminants were localized events that spontaneously faded out (Garin-Bastuji et al., 1990; Ferroglio et al., 1998; Hars and Garin-Bastuji, 2013). However, an unprecedented high seroprevalence (38% in 2013) was reported in the Alpine ibex (*Capra ibex*) population of the Bargy area (French Alps) (Hars et al., 2013; Garin-Bastuji et al., 2014; Mick et al., 2014). This unique situation constitutes the very first case of self-sustained infection with *B. melitensis* in wildlife in Europe. The public health and economic concerns, as well as the conservation issues for this recently restored and protected species, raise many questions in terms of disease management in this population. Determining the drivers of pathogen persistence in this population, in order to further evaluate management strategies, is of the utmost importance. This unique situation has been investigated in-depth, through epidemiological surveys in local populations of ungulates, observation of contacts at the interface with domestic herds and bacterial typing (Mick et al., 2014; Freycon, 2015; Marchand et al., 2017). However, our knowledge of *Brucella* infection and pathogenesis is scant in this species, and cannot be completely extrapolated from knowledge of *B. melitensis* in domestic ruminants. Indeed, in studies on the wild reservoir of *B. abortus* in wildlife in the Greater Yellowstone Area (USA), some pathogenesis characteristics of *B. abortus* were similar between bison (*Bison bison*) and cattle, but specificities such as increased bison susceptibility were also demonstrated (Olsen and Johnson, 2011). Such specificities could also exist in wild Caprinae infected with *Brucella*, but similarities and differences are not currently known.

In the present study, we have addressed the distribution of *Brucella* in organs from seropositive, supposedly infected, ibex, and the consequences in terms of transmission pathways and potential heterogeneity in infectiousness at the individual level in naturally-infected Alpine ibex. Through the bacteriological examination of field or necropsy samples from seropositive ibex, we aimed to: (i) analyze the distribution of *Brucella* in different organs and estimate the frequency of bacterial carriage in seropositive individuals, (ii) identify the different transmission routes, and (iii) study the potential variation of excretion with sex and age. We provide here the first description of *B. melitensis* carriage and shedding in Alpine ibex and discuss the potential consequences of individual heterogeneity in terms of transmission, persistence and disease management in this population.

## Materials and methods

### Study area

Since no case of brucellosis had been reported in domestic ruminants since 2003, France had been declared officially free of ruminant brucellosis (caused by *B. abortus* or *B. melitensis*) by 2005 (Perrin et al., 2016a,b). Nevertheless, in 2012, an outbreak due to *B. melitensis* biovar 3 occurred in a dairy herd in the northern French Alps (Bargy Massif, 46°N, 6.5°E; elevation: 600–2,348 m; area: *ca*. 7,000 ha) (Garin-Bastuji et al., 2014). This re-emergence led to in-depth epidemiological surveys to find the origin of the outbreak. No potential source was identified in other domestic herds (not one infected domestic ruminant out of more than 12,000 tested), suggesting a possible implication of wildlife in this re-emergence. Indeed, an unprecedented high seroprevalence was reported in the Alpine ibex population living in the Bargy area, a remote mountain area. *Brucella* genotyping confirmed that the strain infecting the dairy herd and the ibex was the same, and closely related to the one observed in domestic livestock in 1999 during the last outbreak reported in domestic cattle in the area (Mick et al., 2014). It was thus hypothesized that a probable spillover occurred from domestic animals to ibex in the 90's and a spillback from ibex to domestic cattle in 2011 (ANSES, 2015).

**Figure 1:**
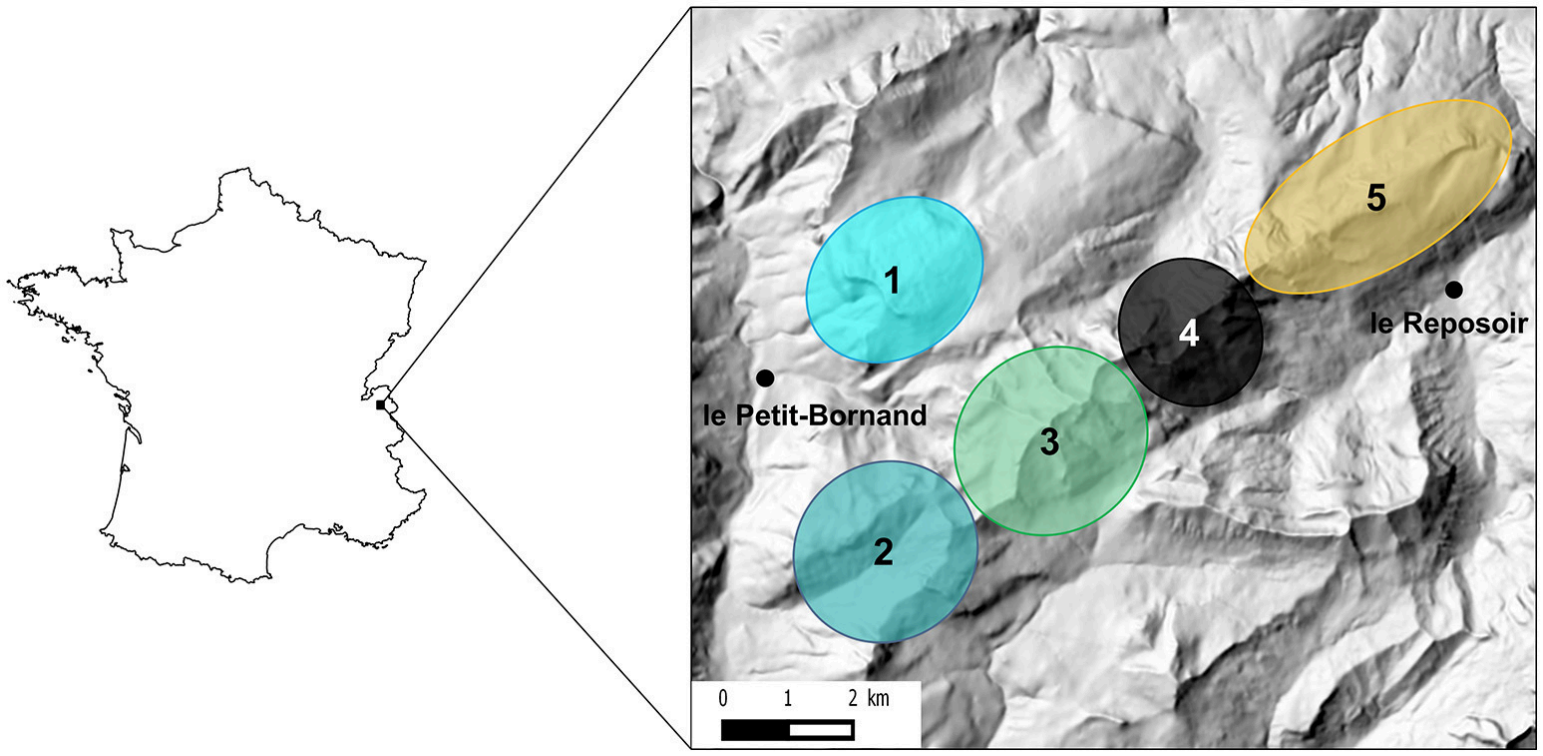
Location of the study area in France and approximate localization of the five socio-spatial units identified in Marchand et al. (2017). These five socio-spatial units correspond to the best number of spatially-segregated groups as determined by hierarchical classification methods on distances between individuals measured as overlap between annual home ranges of GPS-collared female Alpine ibex. See Marchand et al. (2017) for details.

Alpine ibex were reintroduced in the Bargy Massif during two release events in 1974 and 1976 comprising a total of 14 released individuals (Gauthier and Villaret, 1990). Since the discovery of the outbreak in 2012, the Alpine ibex population of the Bargy Massif has been continuously monitored by Capture-Mark-Recapture (CMR). Because there were too few physical recaptures to estimate the population size, data on visual recaptures (capture-mark-resight) were used instead. The population size without newborns was estimated at 567 (95% CI: [487–660]) in 2013, at 310 [275–352] in 2014, and at 277 [220–351] in 2015 (Marchand et al., 2017). Using GPS data on marked individuals, Marchand et al. (2017) demonstrated that females are structured in 5 distinct socio-spatial units (Figure 1), whereas males were prone to move between units especially during the mating period. Noticeable differences in seroprevalence were recorded between these units: 2 units (1 and 2) showed seroprevalences <15%, whereas seroprevalences of the other three spatial units (3, 4, and 5) reached 54, 70, and 35% respectively (Marchand et al., 2017).

### Sampling

A total of 339 animals were captured or recaptured between 2012 and June 2017 (2012–2013: 81, 2014: 71, 2015: 125, 2016: 35, and 2017: 27). Most captures occurred between April and June, corresponding to the last third of pregnancy, on mature animals (≥2 years old). During captures, test-and-cull was implemented on the basis of serological tests (see below for serological methods) as part of management measures decided by the French Authorities (Hars et al., 2013). Seronegative individuals were marked and released while seropositive ones were euthanized, and part of them were collected and necropsied in a biosafety level 3 (BSL-3) facility.

In addition to CMR monitoring and test-and-cull measures, the French Authorities implemented selective culling of animals with observed clinical signs (e.g., presence of visible gross lesions of the joints or the testes, lameness) or individuals older than 5 years, based on the observation that seroprevalence was significantly higher for this age class (Hars et al., 2013). These selective culling operations occurred in spring 2013 (*n* = 5), autumn 2013 (*n* = 233), and spring 2014 (*n* = 18). Non-selective culling on unmarked individuals, considered as having a higher risk of being seropositive than marked individuals (which were all seronegative when released), was also performed in autumn 2015 (*n* = 70). The serological status of these animals was tested using the same assays as for captured animals, when a blood sample of good quality was available.

Carcasses of seropositive animals were collected for necropsy whenever possible, and sex, spatial location and age (estimated by counting horn growth rings, Michallet et al., 1988), were recorded.

Ibex captures were performed by agents and researchers from the French Hunting and Wildlife Agency in accordance with legal and ethical regulations (French environmental code, 2005, 2006; Préfecture de Paris, 2009; Préfecture de la Haute-Savoie, 2013, 2015a,b; French Minister of Ecology Sustainable Development Energy, 2014).

Animals were captured by dart-gun xylazine-ketamine anesthesia (Rompun®, Bayer, Leverkusen, Germany and Imalgène®, Merial, France; 100 mg/individual) (Hars et al., 2013; Marchand et al., 2016, 2017). Seropositive ibex were shot (2012–2013) or euthanized by veterinarians using an embutramide intravenous injection (T61®, Intervet, Angers, France, 2014–2017).

### Serological analyses

All captured animals were blood-sampled by trained technical staff. Four tests were performed in parallel on serum samples:

- The Rose Bengal Test (RBT) and the Complement Fixation Test (CFT), according to requirements of the European Union (EU) for diagnosis of brucellosis in small ruminants and following standards of the World Organization for Animal Health (OIE). For CFT, a cut-off titer of 20 IU/mL was applied according to EU and OIE requirements [EU, 2008; World Organization for Animal Health (OIE), 2016].
- The indirect Enzyme-Linked ImmunoSorbent Assay iELISA (Brucellosis Ovine/Caprine Ab Test, IDEXX, Montpellier, France) and the blocking cELISA (INgezim Brucella Compac 2.0, Ingenasa, Madrid, Spain).

In 2012–2013, individuals considered as seropositive on the basis of at least two of the four above-mentioned tests were shot after laboratory testing. In 2014, a rapid Laminar Flow Immune-chromatographic Assay (LFIA) (Rapid G.S. Brucella Ab test, Bionote, Gyeonggi-do, Rep. of Korea) had been validated on ibex samples by the EU/OIE/FAO and National reference laboratory (ANSES, 2014; Corde et al., 2014). Results showed a very good correlation in laboratory conditions between the LFIA and the other four tests on serum samples from 2012–2013. Moreover, a trial in field conditions confirmed the reliability of the LFIA. Therefore, since 2014, Ibex were euthanized when positive to LFIA, and their serostatus was further confirmed in the laboratory using the same four tests as in 2012–2013 and considered as seropositive when at least two of the laboratory tests were positive.

### Bacterial culture (for *brucella*)

For each necropsied animal, bacterial cultures were performed on 1–15 organs, as the list of organs selected for systematic bacteriological examination was only standardized in 2016. Statistical analyses were shaped to take into account this heterogeneity in the bacteriological examination between individual ibex (section Statistical Analyses).

This list comprised organs that may have shedding potential (e.g., testes, genital tract, supramammary, internal iliac and inguinal lymph nodes, urine and/or bladder udder), organs with lesions compatible with brucellosis (e.g., gross lesions of the joints or the testes, abscesses) and fetuses. Fetuses were analyzed independently from their mother on heart, kidneys, spleen, abomasum, liver, and testes. Other organs (such as spleen, cotyledons, and amniotic fluid) had been sampled on some ibex necropsied by 2013–2015, to better characterize *Brucella* distribution. Finally, genital swabs (either vaginal or preputial) were performed in the field (before necropsy) and were used for bacterial cultures in some individuals in 2013–2015.

Tissues were sampled during necropsy, and either directly analyzed by culture, or frozen for delayed culture. Cultures were performed by a local laboratory officially authorized to provide brucellosis diagnoses (BSL-3 facilities): the veterinary laboratory of the Savoie department (LDAV 73). Isolated *Brucella* strains were further bio-typed by the National Reference Laboratory for animal brucellosis (ANSES). All methods were performed according to OIE standards as described previously (Alton et al., 1988; Mick et al., 2014; World Organization for Animal Health (OIE), 2016). Positive cultures were semi-quantitatively enumerated [1: <10 colony-forming units (CFU), 2: 10–50 CFU, 3: 50–100 CFU, 4: > 100 CFU]. The raw data supporting the conclusions of this manuscript will be made available by the authors, without undue reservation, to any qualified researcher.

### Definition of organ categories and ibex infection status

We separated the analyzed organs into three categories in relation to their shedding potential:

- Urogenital organs (testes, genital tract, genital swab in the field, urine or bladder) can directly excrete the bacteria in the environment;
- Lymph nodes from the pelvic area (supramammary, internal iliac and inguinal lymph nodes), that drain the udder and the reproductive organs and can reflect a latent infection with potential further urogenital or milk excretion following recirculation of the bacteria;
- Other “entry” or “closed” organs, such as retropharyngeal lymph nodes, joints, visceral abscesses, which reflect the presence of the bacteria but with a much lower risk of excretion.

Since the risk of bacterial shedding varied according to the category of organ infected, we considered four infection statuses in ibex according to the previous organ categories and positive results from culture (Table 1). Individuals having at least one positive culture in any organ were considered as actively infected. Among them, animals being actively infected in organs in the pelvic area (at least one positive culture for organs in the pelvic area, i.e., urogenital samples or lymph nodes from the pelvic area) were considered as having a risk of potential shedding during present or future reproductive cycles. Finally, among them, individuals actively infected in urogenital organs (having at least one positive culture for the urogenital samples) were considered as having a risk of potential shedding at the time of capture. Individuals with negative bacterial cultures were called “bacteriologically unconfirmed” but could have been misdiagnosed, since bacteriological examination is not a method with 100% diagnostic sensitivity.

**Table 1:** Definition of infection classes for *Brucella melitensis* in Alpine ibex, depending on bacteriological results on tissue samples of the three categories of organ (+: at least one positive culture, –: all cultures negative).

**Individual status**			**Urogenital samples**	**Lymph nodes in the pelvic area**	**Other organs**
Bacteriologically confirmed (active infection in any organ)	Active infection in organs in the pelvic area	Active infection in urogenital organs	+	±	±
			–	+	±
			–	–	+
Bacteriologically unconfirmed			–	–	–

### Statistical analyses

Statistical analyses were performed using the R software version R 3.4.1 (R Core Team, 2017). We first intended to analyze the probability of an individual being actively infected in any organ, in organs in the pelvic area, or in urogenital organs (i.e., fetuses were not included in these analyses). However, the number of bacterial cultures varied among individuals, thus the probability of finding at least one positive culture in an ibex depended on the number of cultures assayed. To take into account this unequal number of samples between individuals, we used grouped binary data for each individual, where “successes” and “failures” were the numbers of positive and negative bacteriological results, respectively. The dependent variable was thus the probability of observing a positive bacteriological result on a panel of examined organs, using the number of organs tested per individual as weights. Therefore, we analyzed these data using a generalized linear model with a binomial distribution (Zuur et al., 2009), and in order to account for random/cluster effects in our dataset, we used a mixed model with glmer from the *lme4* R package, with the spatial unit and the year fitted as random effects (Bates et al., 2015). The binomial generalized linear mixed model expresses the log of the odds of the probability of a bacterial culture being positive as a linear function of the explanatory variables. For each explanatory variable, the odds ratio (OR) was therefore expressed as the exponential of the associated model coefficient (Dohoo et al., 2009).

Three models were adjusted to analyze the probability of a bacterial culture being positive in (i) any organ, (ii) organs in the pelvic area, or (iii) urogenital organs. According to its capture or culling location, each individual was assigned to one of the five socio-spatial units structuring the population previously identified by Marchand et al. (2017). To control for possible variation in the epidemiological background among these socio-spatial units, we considered them as a random effect (ZONE) and we excluded from analyses one individual for which the capture location was missing. Because of small sample size, we also merged individuals belonging to units 1 and 2, since both have similar low seroprevalences. We tested for the influence of sex (SEX) and age (continuous variable in years, AGE) as fixed effects. We tested the influence of the CFT titer as a fixed effect (TITER) as a proxy for active infection, because high levels of circulating antibodies have been linked to active *Brucella* infection in the domestic species (Durán-Ferrer et al., 2004). As longitudinal studies in Alpine ibex infected by *Brucella* are not easily conceivable, we could not test this hypothesis, but we assumed that this was also the case in this species. We excluded one individual with a negative CFT (<20 UI/mL), and six animals on which CFT was not performed. We also tested for the period of sampling as a fixed effect (before/after the mass culling performed in autumn 2013: PERIOD). Indeed sampling biases were likely in 2012–2013 (captures focusing on animals with the most obvious clinical signs or gross lesions—Freycon et al., 2017). We also used the year of capture as a random effect (YEAR) to control for possible sampling biases and varying epidemiological situations among years (within periods), that could lead to varying shedding patterns. Finally, we tested whether freezing of samples/tissues would decrease the sensitivity of subsequent bacterial cultures by decreasing the amount of *Brucella*. To this end, we also considered sample treatment as a fixed effect (fresh or frozen: TREATMENT). We also tested for the effect of first-order interactions between sex, age, and CFT because the course of *Brucella* infection may differ between males and females (Zuk and McKean, 1996).

The semi-quantitative assessment of bacterial load also allowed us to analyze the variations in *Brucella* carriage among organs, to test whether *Brucella* preferentially infected specific organs and whether this preference varied with age or sex. This expected heterogeneity would have direct implications on the shedding potential, as we assume that the higher the bacteria load in organs, the higher the shedding probability in associated regions (genital, inguinal, mammary) or fluids (urogenital secretions, semen, fetal fluids). After examining the variable distribution, we used a GLMM with a negative binomial distribution (glmmadmb function from the *glmmADMB* R package, Fournier et al., 2012; Skaug et al., 2013), where the response variable was the semi-quantitative assessment of the number of CFU per plate for each tissue sample. Since several samples originated from the same individual, we used individual identity (ID) as a random effect. We included the same fixed additive effects as before and added the organ category to evaluate potential differences in *Brucella* distribution in the organism (ORGAN). We also tested the first order interactions between sex, age, CFT titer and organ category in order to assess the variation over time of *Brucella* distribution in each sex.

For all analyses, after adjusting the complete model, we compared all possible sub-models using the dredge function (*MuMIn* R package, Kamil, 2016). Model selection was performed using the Akaike Information Criterion corrected for small sample size (AIC_c_), keeping models for which the difference between their AIC_c_ value and the lowest AIC_c_ value (ΔAIC_c_) was <2 (Burnham and Anderson, 2002). For each candidate model, we examined the Akaike weights (W), which are relative model likelihoods normalized over the likelihoods of all possible sub-models. A weight can be interpreted as the probability for a candidate of being the best model given the data and the set of possible sub-models (Wagenmakers and Farrell, 2004). We chose the final model based on the number of parameters, following the principle of parsimony: we chose the model with the fewest parameters from the set of “best models” and when two models had the same number of parameters, we chose the model with the highest Akaike weight.

On the final model, we verified that random effects were normally distributed, and checked adequacy of the model with residual plots. The amounts of variability explained by the fixed and random effects of generalized linear mixed models were respectively estimated using marginal and conditional *R*2 of Nakagawa and Schielzeth (2013).

## Results

### Collected data

Bacterial cultures were performed on 88 seropositive individuals slaughtered between October 2012 and June 2017: 56 females aged from 2 to 14 (pregnant: 11, non-pregnant: 42, unknown: 3) and 32 males aged from 2 to 15. The sampling was heterogeneous among spatial units (Units 1–2: 6, Unit 3: 41, Unit 4: 23, Unit 5: 17, unknown: 1) and over time (2012: 11, 2013: 20, 2014: 30, 2015: 16, 2016: 5, 2017: 6). Among the 88 individuals, 81 were necropsied at the LDAV73, whereas only genital swab and blood sampling was performed on the seven remaining animals. Most individuals were captured or culled in spring during the last third of gestation (43 females, 28 males), and a few were culled or captured in autumn before the mating period (13 females, 4 males).

Among the 88 seropositive ibex, one was negative with RBT and CFT (<20 UI/mL) but was positive on LFIA, iELISA and cELISA; one was negative in RBT but positive on LFIA, CFT, iELISA, and cELISA; one was negative in iELISA but positive on LFIA, RBT, CFT, and cELISA; and six individuals culled in 2015 were only tested by LFIA because of small blood samples; all six LFIA tests were positive. The 79 remaining ibex were positive to all tests performed and did not show inconsistency between tests. Overall, CFT titer decreased with age in both males and females (data not shown).

**Figure 2:**
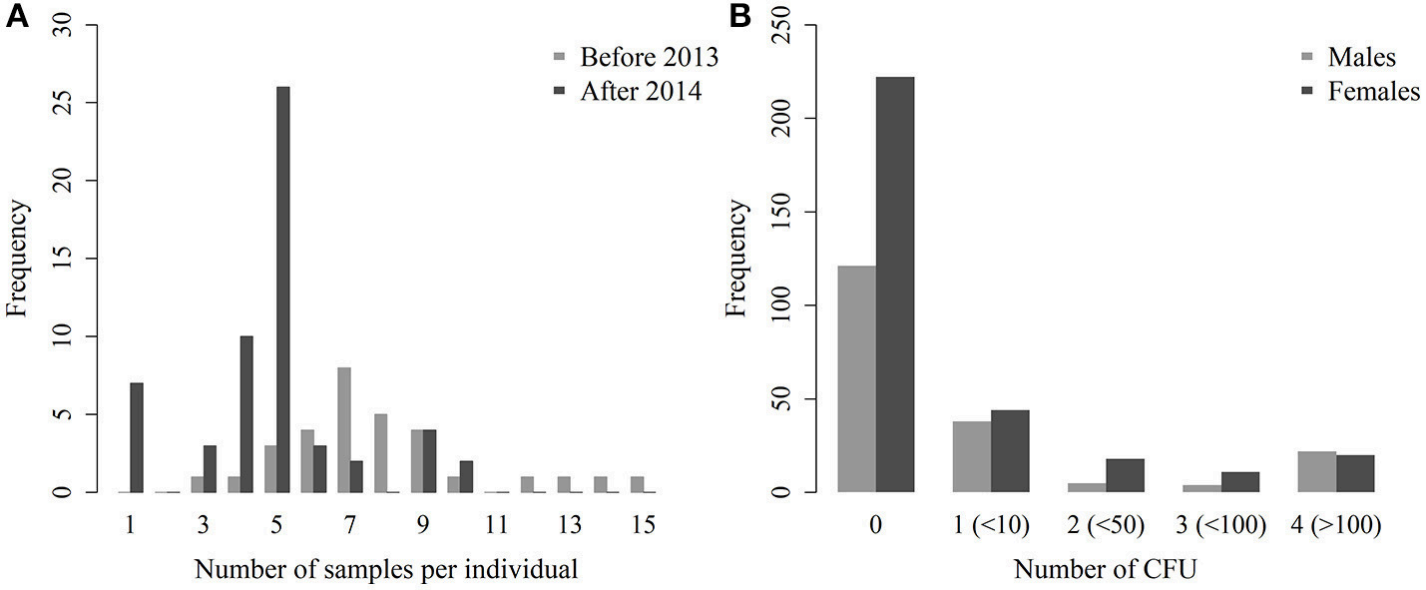
Distribution of the number of samples per individual, before and after the culling implemented in 2013 **(A)** and number of CFU per plate for males and females **(B)**.

A total of 516 bacteriological results were obtained from these 88 animals. The number of analyzed organs per individual ranged from 1 to 15 (mean: 5.9, 95% CI: [5.3–6.4]—Figure 2A). In 2012–2013, the number of analyzed samples per individual was higher (7.8 [6.8–8.8]) than during the following years (4.8 [4.2–5.4]). Out of these 516 bacterial cultures, 173 tissues/samples, were positive (1–9 positive tissues/samples per individual, 2.0 on average [1.4–2.5]). The number of analyzed organs and the number of positive results are shown in detail in Table 2. Positive abscesses and positive samples from other organs came from the spleen (*n* = 6), udder (5), other lymph nodes (5), lungs (4), nuchal ligament (2), C3/C4 cervical vertebrae (1), cotyledons (1), and mesentery (1).

**Table 2:** Number of bacterial cultures performed in Alpine ibex between October 2012 and June 2017 in search for *Brucella melitensis*, and number of positive cultures for each tissue sample of three categories of organs.

**Organ category**	**Tissue sample**	**Females**	**Males**
Urogenital samples	Genital swab (in-the-field)	6/36 (17%)	5/23 (22%)
	Genital tract	9/41 (22%)	5/11 (45%)
	Urine or bladder	3/16 (19%)	5/15 (33%)
	Testes	–	12/32 (37%)
Lymph nodes in the pelvic area	Supramammary lymph nodes	17/48 (35%)	–
	Inguinal lymph nodes	1/1	6/19 (32%)
	Internal iliac lymph nodes	18/50 (36%)	12/29 (41%)
Other organs	Retropharyngeal lymph nodes	14/47 (30%)	12/28 (43%)
	Joints	11/19 (58%)	12/21 (57%)
	Abscesses	6/6	3/4
	Other organs	14/57 (25%)	2/13 (15%)
Total		99/321 (31%)	74/195 (38%)

*One to15 samples were cultured per individual, leading to 516 bacteriological results from 88 animals. Percentages of positive cultures are indicated in brackets except when the sample size was <10*.

The semi-quantitative evaluation of the number of CFU was available for all but 11 positive culture results. These 11 positive cultures were therefore excluded from the model analyzing the number of CFU in each organ. Among the 505 remaining tissues/samples, the mean semi-quantitative evaluation of the number of CFU was 0.6 in females (95% CI: [0.5–0.7]) and 0.8 in males ([0.6–1.0]—Figure 2B). In positive tissues/samples, the mean semi-quantitative number of CFU was 2.1 ([1.9–2.3]), meaning that there were between 10 and 50 CFU on average.

When gross lesions of the joints were present, associated bacterial culture of the joint fluid was positive in 17 cases, negative in 15 cases. On the other hand, six bacterial cultures were positive and two negative for joints without gross lesions. Bacterial cultures of joints with gross lesions were not significantly more often positive than those of joints without gross lesions (Fisher's exact test, *p* = 0.428). Similarly, when gross lesions of the testes were present, bacterial culture was positive in six cases and negative in three. In the absence of gross lesions, six bacterial cultures were positive and 15 were negative. To conclude, cultures of testes in the presence of gross lesions were not significantly more often positive than in the absence of gross lesions (Fisher's exact test, *p* = 0.102).

Bacteriological results were also obtained for three fetuses, two males and one female, for which four to six organs were analyzed: heart (*n* = 2), kidneys (*n* = 3), liver (*n* = 3), spleen (*n* = 2), abomasum (*n* = 3), and testes (*n* = 2). One of these fetuses was positive for all analyzed organs (heart, kidneys, liver, spleen, and abomasum). Its mother was also positive for 8 out of 9 of the analyzed organs, including cotyledons (Table 3). The other two fetuses were negative for all analyzed organs, while their mothers were positive for 3 out of 10 and 1 out of 9 analyzed organs respectively. Cotyledons were negative for both, as well as for four other females analyzed in previous years.

**Table 3:** Results of bacteriological detection of *Brucella melitensis* on three fetuses of Alpine ibex and their mothers in 2017.

	**Organ category**	**Tissue sample**	**Fetus#1**	**Fetus#2**	**Fetus#3**
Fetuses		Heart	4	–	0
		Kidneys	4	0	0
		Liver	1	0	0
		Spleen	4	–	0
		Abomasum	4	0	0
		Testes	–	0	0
Total (positive/analyzed)			5/5	0/4	0/6
Mothers	Urogenital samples	Genital swab (in-the-field)	–	–	–
		Genital tract	–	–	–
		Urine or bladder	4	0	0
	Lymph nodes in the pelvic area	Supramammary lymph nodes	2	2	0
		Inguinal lymph nodes	–	–	–
		Internal iliac lymph nodes	4	1	0
	Other organs	Retropharyngeal lymph nodes	4	0	0
		Joints	0	0	0
		Abscesses	–	2 (udder)	–
		Spleen	4	0	0
		Lungs	4	0	0
		Cotyledons	4	0	0
		Superficial cervical (prescapular) lymph nodes	4	–	3
		Other	–	0	–
Total (positive/analyzed)			8/9	3/10	1/9

*0, negative bacterial culture; 1–4, positive bacterial culture (1: <10 CFU, 2: 10–50 CFU, 3: 50–100 CFU, 4: >100 CFU)*.

### Bacterial distribution and shedding potential

Among the 88 seropositive animals (fetuses excluded), 37 (42%) were negative for all analyzed samples and thus infection was not bacteriologically confirmed. The remaining 51 (58%), which were positive for at least one sample, were considered as having active infection. Among them, 45 were positive for at least one lymph node from the pelvic area (i.e., active infection in the pelvic area), including 26 positives for at least one urogenital sample (i.e., active infection in urogenital organs, Table 4). In seropositive females, the proportion of active infection in organs in the pelvic area was 45% (25/56), and 21% in urogenital organs (12/56). Among the 32 seropositive males, 20 (62%) were actively infected in organs in the pelvic area, and 14 (44%) were actively infected in urogenital organs (Table 4).

**Table 4:** Distribution of infected Alpine ibex according to classes established from the category of organs positive for *Brucella melitensis* and to the number of positive samples per individual.

**Organ category**	**Number of positive samples**	**Bacteriologically unconfirmed** **(*****n*** = **37)**		**Active infection in any organ** **(*****n*** = **51)**		**Active infection in organs in the pelvic area** **(*****n*** = **45)**		**Active infection in urogenital organs** **(*****n*** = **26)**	
		**♂**	**♀**	**♂**	**♀**	**♂**	**♀**	**♂**	**♀**
Urogenital samples	0	27	10	17	8	13	6	0	0
	1	–	–	7	9	7	9	7	9
	2+	–	–	5	5	5	5	5	5
LN in the pelvic area	0	27	10	6	9	2	7	2	7
	1	–	–	10	8	10	8	4	4
	2+	–	–	13	5	13	5	6	3
Other organs	0	27	10	7	7	7	7	4	3
	1	–	–	13	7	10	5	2	5
	2+	–	–	9	8	8	8	6	6

*See Table 1 and text for definition of categories (bacteriologically unconfirmed, active infection in any organ/in organs in the pelvic area/in urogenital organs). Actively infected individuals in any organ include actively infected individuals in organs in the pelvic area and in urogenital organs*.

When at least two organs were analyzed, the majority of bacteriologically confirmed ibex (active infection in any organ) had more than one positive sample (37/49 i.e., 76%, Table 4).

### Probability of positive culture in a panel of organs

**Figure 3:**
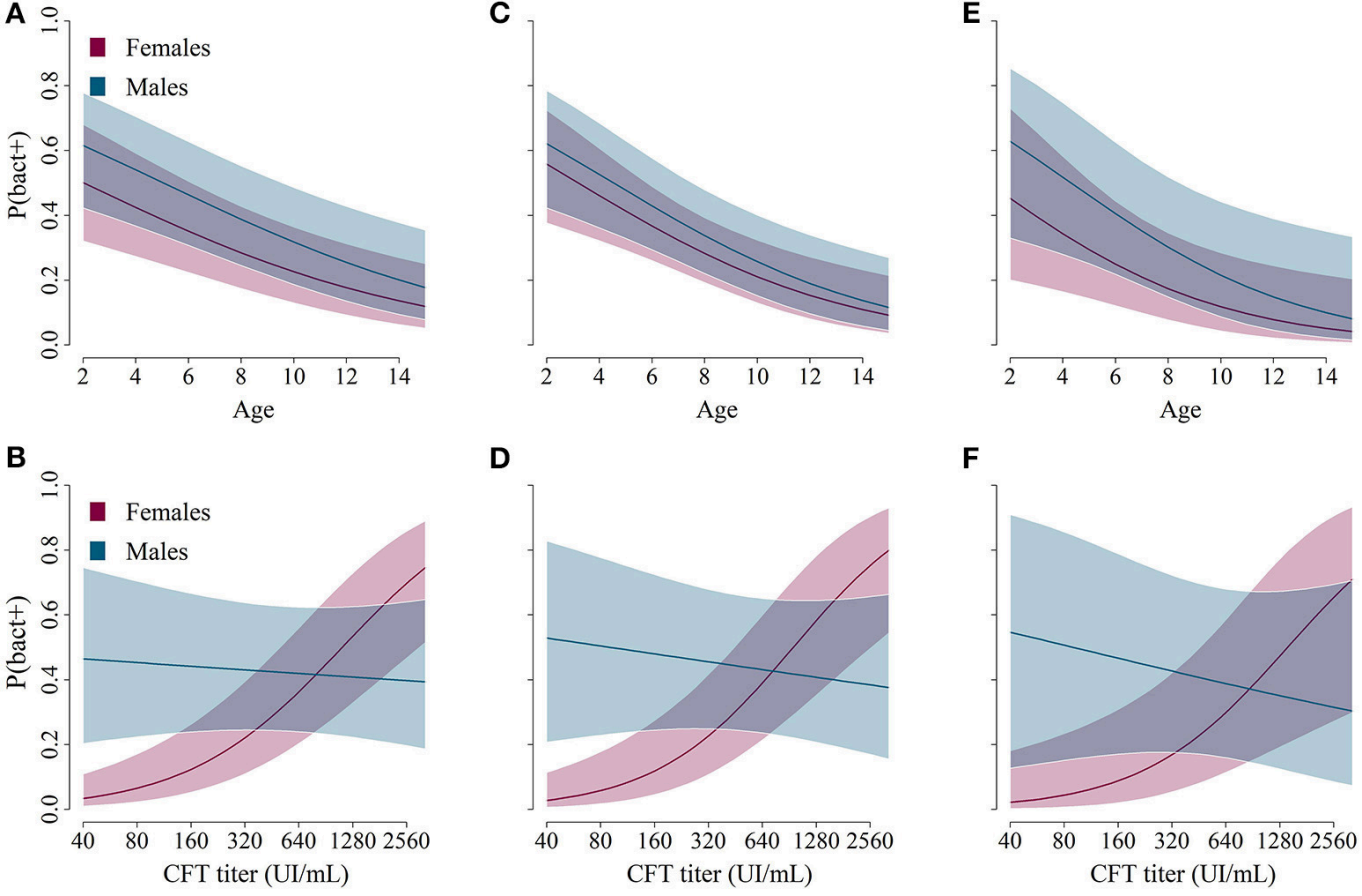
Predictions from the best models describing the relationship between age/sex **(A,C,E**, on the top) or complement fixation titers/sex **(B,D,F**, on the bottom) and the predicted probability for ibex of belonging to a specific class: bacteriologically confirmed **(A,B**, left: *Brucella* found in any organ), active infection in organs in the pelvic area **(C,D**, middle: *Brucella* found in urogenital samples and/or lymph nodes in the pelvic area), or active infection in urogenital organs **(E,F**, right: *Brucella* found in urogenital samples).

The model best fitting the probability of a bacterial culture being positive in any organ included the variables AGE, SEX, TITER and the interaction SEX:TITER. These variables were present in all models with ΔAICc<2 (Table 5). The probability of a bacterial culture being positive decreased significantly with increasing age (odds ratio OR_per year_ = 0.85, 95% CI: [0.77–0.93]—Table 6 and Figure 3A), increased with increasing TITER for females (OR_per 1 unit in log(TITER)_ = 2.71 [1.84–3.97]) but not for males (OR_per 1 unit in log(TITER)_ = 0.83 [0.45–1.52]—Table 6 and Figure 3B), and tended to be higher for males than for females (OR_males vs. females_ = 1.55 [0.97–2.47]). It is important to note that, infection being generally active in young individuals, the CFT titer decreased with age (OR_per year_ = 0.85 [0.79–0.91], *p* < 0.001). Consequently, a confounding effect was possible between the effects of AGE and TITER, however both variables still appeared in the selected model.

**Table 5:** Model selection table to analyze the probability of positive bacterial culture for any organ, for organs in the pelvic area, or for urogenital samples (random variables: ZONE and YEAR), and to analyze the number of CFU in each organ for females and for males (random variable: ID).

**Model**	**DF**	**LL**	**AICc**	**Δ**	**W**
**PROBABILITY OF POSITIVE BACTERIAL CULTURE FOR ANY ORGAN**					
**AGE + SEX × log(TITER)**	**7**	−**154.92**	**325.4**	**0.00**	**0.196**
AGE + SEX × log(TITER) + AGE:log(TITER)	8	−154.05	326.1	0.73	0.136
AGE + SEX × log(TITER) + AGE:log(TITER) + TREATMENT	9	−153.01	326.6	1.20	0.107
AGE + SEX × log(TITER)+TREATMENT	8	−154.37	326.8	1.38	0.098
AGE + SEX × log(TITER) + PERIOD	8	−154.61	327.2	1.84	0.078
**PROBABILITY OF POSITIVE BACTERIAL CULTURE FOR ORGANS IN THE PELVIC AREA**					
**AGE + SEX × log(TITER)**	**7**	−**112.66**	**240.9**	**0.00**	**0.271**
AGE + SEX × log(TITER) + PERIOD	8	−112.27	242.6	1.71	0.115
AGE + SEX × log(TITER) + AGE:log(TITER)	8	−112.37	242.8	1.90	0.105
**PROBABILITY OF POSITIVE BACTERIAL CULTURE FOR UROGENITAL ORGANS**					
**AGE + SEX × log(TITER) + TREATMENT**	**8**	−**69.01**	**156.1**	**0.00**	**0.124**
AGE + SEX × log(TITER) + TREATMENT + PERIOD	9	−67.81	156.2	0.14	0.115
AGE + SEX × log(TITER) + TREATMENT + AGE:SEX	9	−68.43	157.4	1.37	0.062
AGE + SEX × log(TITER) + TREATMENT + PERIOD + AGE:SEX	10	−67.17	157.5	1.46	0.060
AGE + SEX × log(TITER) + AGE:SEX	8	−70.00	158.0	1.98	0.046
**NUMBER OF CFU IN EACH ORGAN**					
SEX × ORGAN + log(TITER) + SEX:log(TITER) + AGE	11	−477.79	978.2	0.00	0.071
SEX × ORGAN + log(TITER) × AGE + SEX:log(TITER)	12	−476.93	978.5	0.38	0.058
**SEX × ORGAN + log(TITER) + SEX:log(TITER)**	**10**	−**479.10**	**978.7**	**0.53**	**0.054**
SEX × ORGAN + log(TITER) + SEX:log(TITER) + AGE + AGE:SEX	12	−477.42	979.5	1.37	0.036
SEX × ORGAN + log(TITER) + SEX:log(TITER) + AGE + PERIOD	12	−477.52	979.7	1.56	0.032
SEX × ORGAN + log(TITER) × AGE+SEX:log(TITER) + PERIOD	13	−476.51	979.8	1.67	0.031
SEX × ORGAN + log(TITER) + SEX:log(TITER) + AGE + TREATMENT	12	−477.65	980.0	1.82	0.028
SEX × ORGAN + log(TITER) × AGE + SEX:log(TITER) + TREATMENT	13	−476.63	980.1	1.91	0.027

*For each candidate model, the table gives the fixed variables (see text for definition), the number of Degrees of Freedom (DF), the Log-Likelihood (LL), the Akaike Information Criterion corrected for small sample size (AICc), ΔAICc<2 (Δ), and the Akaike Weight (W, see text for definition). The model selected is in bold*.

**Table 6:** Parameters of selected models to explain the probability of positive bacterial culture for any organ, for organs in the pelvic area, or for urogenital samples, and to explain the number of CFU in each organ.

**Response variable**	**Explanatory variable and modality**	**OR and 95% confidence interval**	***P*-value of Wald test**
Probability of positive bacterial culture for any organ	AGE	0.85 [0.77–0.93]	<0.001
	SEX (males)	1.55 [0.97–2.47]	0.066
	log(TITER)	2.71 [1.84–3.97]	<0.001
	SEX:log(TITER)	0.31 [0.19–0.49]	<0.001
Probability of positive bacterial culture for organs in the pelvic area	AGE	0.84 [0.74–0.95]	0.004
	SEX (males)	1.49 [0.82–2.70]	0.189
	log(CFT)	2.82 [1.70–4.67]	<0.001
	SEX:log(TITER)	0.27 [0.14–0.51]	<0.001
Probability of positive bacterial culture for urogenital samples	AGE	0.80 [0.66–0.97]	0.025
	SEX (males)	1.62 [0.65–4.05]	0.304
	log(TITER)	2.87 [1.30–6.30]	0.009
	SEX:log(TITER)	0.21 [0.08–0.57]	0.002
	TREATMENT (fresh)	3.24 [1.11–9.43]	0.031
Number of CFU in each organ	SEX (males)	3.69 [1.51–9.03]	0.004
	ORGAN (NLpelvic)	2.33 [1.32–4.11]	0.004
	ORGAN (others)	2.65 [1.53–4.59]	<0.001
	log(TITER)	2.37 [1.43–3.94]	<0.001
	SEX(males):ORGAN(NLpelvic)	0.45 [0.20–1.00]	0.050
	SEX(males):ORGAN(others)	0.33 [0.16–0.69]	0.003
	SEX:log(TITER)	0.39 [0.19–0.80]	0.010

*The reference levels are females (for the variable SEX), frozen (for the variable TREATMENT), and urogenital (for the variable ORGAN)*.

The AGE:TITER interaction (OR = 1.07 [0.97–1.17]), the TREATMENT variable (OR_fresh vs. frozen_ = 1.37 [0.76–2.47]) and the PERIOD variable (OR_2012–2013 vs. 2014–2017_ = 1.79 [0.43–7.51]) were also present in some of the selected models based on the AICc, but they did not significantly improve the model-data fit and were thus not retained in the most parsimonious model (Table 5).

Regarding the probabilities of positive culture in pelvic area organs and of positive culture in urogenital organs, both models retained the variables AGE, SEX, TITER, and the interaction SEX:TITER, and the results were qualitatively and quantitatively the same as before (Tables 5, 6 and Figures 3C–F). Unlike the other two, the best model for the probability of a bacterial culture being positive in urogenital organs also included the variable TREATMENT, which was present in all models with ΔAICc<2 but one. Fresh samples had higher probabilities of being culture-positive than frozen ones (OR = 3.24 [1.11–9.43]). The interaction AGE:SEX (OR = 1.31 [0.94–1.81]) was also present in some models with ΔAICc<2 (Table 5) but was not retained in the final model.

### *Brucella* carriage in organs

**Figure 4:**
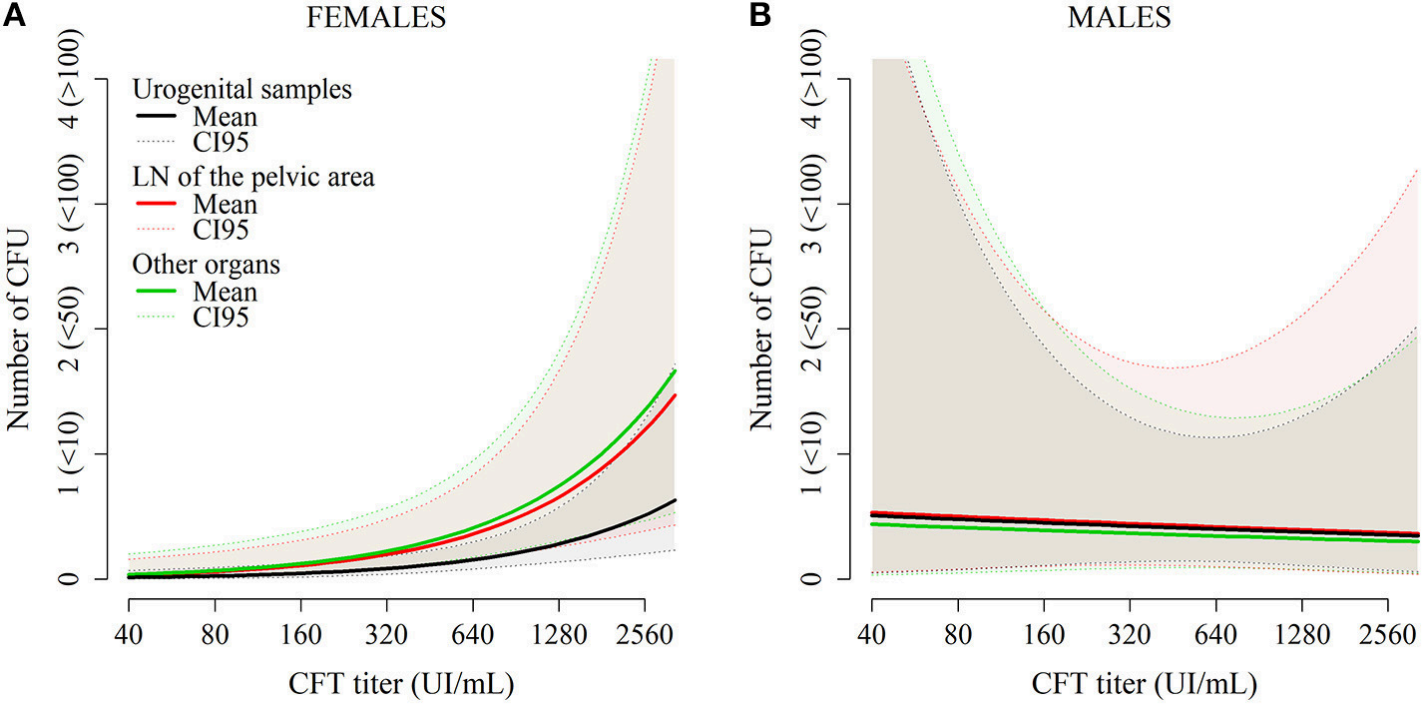
Predictions from the model describing the number of CFU per plate for each organ in females **(A)** and in males **(B)** as a function of CFT titers and organ category.

Regarding bacteria carriage on the organ level, the final model included the SEX, ORGAN, TITER variables, and the SEX:ORGAN and SEX:TITER interactions. These variables were present in all models with ΔAICc<2 (Table 5). The average bacterial load was significantly higher in males than in females (OR = 3.69 [1.51–9.03]). In females, the urogenital organs had lower bacterial loads compared to lymph nodes from the pelvic area (OR = 2.33 [1.32–4.11] compared to urogenital organs) or to other organs (OR = 2.64 [1.53–4.59] compared to urogenital organs, Table 6 and Figure 4A). In males, bacterial loads were not significantly different between organ categories (OR = 1.05 [0.39–2.80] for lymph nodes from the pelvic area and OR = 0.86 [0.38–2.23] for other organs—Figure 4B). The bacterial load increased with increasing TITER for females (OR = 2.37 [1.43–3.94]) but not for males (OR = 0.92 [0.38–2.23]).

The AGE (OR_per year_ = 0.90 [0.79–1.02]), TREATMENT (OR_fresh vs. frozen_ = 1.23 [0.58–2.63]), PERIOD (OR_2012−2013 vs. 2014−2017_ = 1.38 [0.59–3.25]) variables and the AGE:SEX (OR = 0.89 [0.67–1.17]) and AGE:TITER (OR = 1.09 [0.96–1.24]) interactions were also present in models with low AICc values, but not in the most parsimonious model.

## Discussion

This study analyzes the largest dataset of bacteriological investigations on Alpine ibex infected by *Brucella* ever collected to date. Using semi-quantitative measurements of bacterial load in a large variety of organs allowed us to infer the pattern of bacteria shedding in infected animals as well as its variation among individuals, and to explore the level of active infection in seropositive individuals. In summary, the probability of bacteria presence decreased with ibex age, in both males and females. For a given age, females with highest CFT titers exhibited the highest bacteria presence and load, a trend not observed in males. The average bacterial load was significantly lower in females than in males. Furthermore, in females, pathogen loads were lower in urogenital organs than in other organs, whereas no differences were observed between organ categories in males. We also reported the presence of *Brucella* at high bacterial load in fetuses in one out of three and in cotyledons of one out of seven necropsied females in the last third of pregnancy, confirming that pregnancy represents a particular at-risk situation for both horizontal and vertical transmission, as it does in domestic ruminant species.

Overall, our results revealed (i) a high probability of detecting *Brucella* in seropositive ibex (58% on average), (ii) a broad bacterial dissemination and high *Brucella* carriage both at individual and tissue/sample levels (2.0 infected tissues/samples per individual and around 10–50 CFU per infected tissues/samples on average), and (iii) a strong variation in the bacteriological carriage and shedding capacity according to ibex sex, age, and serological status. In the following sections, we will discuss each of these results, each time highlighting their limitations and inferring on the epidemiology of the infection in Alpine ibex.

### Prevalence of bacterial detection

We selected supposedly infected ibex based on serological test results. The diagnostic sensitivity of serological tests are good in domestic animals, i.e., up to 97% in cattle (Godfroid et al., 2010, and references therein) and up to 100% in sheep and goats (European Commission, 2001, and references therein), but are still not well-defined in ibex. Even though the serological reaction of ibex seems strong and long-lasting, a lack of sensitivity cannot be excluded, which might represent a limit to our results by missing some infected animals, especially young sexually immature or old immunosenescent individuals. One possible option would have been to necropsy and perform bacterial cultures on seronegative individuals also, which was unfortunately not performed given many legal restrictions (mainly seropositive animals were euthanized), as well as practical and costs aspects (transportation of large carcasses from remote mountains areas, huge necropsy, and bacteriological effort allowing only the analysis of some carcasses, therefore the choice was made to target seropositive individuals for investigating bacterial carriage). It should be noted, however, that six bacterial cultures were performed on four entirely seronegative individuals from the same area, and that 17 bacterial cultures were performed on three found-dead animals on which serological tests could not be performed. All 23 bacterial cultures were negative (data not shown).

To detect active infection, we chose bacterial culture, the gold standard for brucellosis diagnosis [Alton et al., 1988; World Organization for Animal Health (OIE), 2016]. We targeted organs that were described as the preferred tissues for bacteria isolation, i.e., retropharyngeal, supramammary, iliac and inguinal lymph nodes, as well as genital organs and vaginal secretions (Marín et al., 1996; European Commission, 2001; Godfroid et al., 2013). Because of obvious limitations in terms of time and costs, we were not able to necropsy all seropositive animals culled since the discovery of the outbreak, or perform bacterial cultures in all relevant organs from all individuals. In particular, the list of organs selected for systematic bacteriological examination was only standardized in 2016, which represents another limit of our study. In future studies, variability between samples would be reduced, if the standard protocol implemented since 2016 is still followed.

*Brucella* were not detected in 42% of seropositive individuals; these animals probably cleared the bacteria or had a low *Brucella* carriage in organs undetectable by the bacterial culture at the time of capture. Other techniques such as the quantitative polymerase chain reaction (qPCR) assay have been developed (Yu and Nielsen, 2010), but, up to now, there is no report of higher diagnostic sensitivity of a particular PCR method compared with bacterial culture (Godfroid et al., 2013). In particular, PCR has been demonstrated as inaccurate to detect all culture-positive animals in bison (Treanor et al., 2011). However, qPCR could have allowed us to improve diagnosis sensitivity, in particular if used in parallel with the bacterial culture, by detecting the DNA of the bacteria, even in samples with dead or very few live *Brucella*.

Even if the proportion of 58% of ibex with at least one positive bacterial result may be underestimated in our sample, this result remains high given the significance of the presence of live *Brucella* (including in terms of zoonotic risk). Proportion of animals with active infection in naturally-infected seropositive cattle was under 50% in several studies [e.g., 46% of 355 cattle in Harrington and Brown (1976), 49.2% of 2,570 cattle in Huber and Nicoletti (1986) or 47.6% of 21 cattle in O'Leary et al. (2006)], as it was in wild bison (46% of 26 bison—Roffe et al., 1999). In small domestic ruminants, proportions were more contrasted, with a proportion of 62.2% of actively infected animals among 45 naturally-infected seropositive sheep (Ilhan et al., 2008), whereas a lower proportion has been found in naturally-infected goats (1 out of 12 seropositive goats—Ribeiro et al., 1990).

Our data supports a high shedding potential in ibex infected by *B. melitensis*, which may be a major factor of a self-sustained enzootic transmission. This could be explained by a high susceptibility of ibex to brucellosis. Specific differences in susceptibility to brucellosis have already been demonstrated between bison and cattle, with bison being more susceptible to the disease and getting infections in fetuses, the uterus or mammary glands more often (Olsen and Johnson, 2011). Moreover, the genetic diversity of reintroduced Alpine ibex populations is typically low, which can affect susceptibility to infection (Biebach and Keller, 2010), and this could explain, at least in part, the high susceptibility of the Alpine ibex population of the Bargy Massif to *B. melitensis*. In the future, experimental studies aiming at determining the relative susceptibility of ibex as compared to domestic species and other wild species such as Alpine chamois (*Rupicapra rupicapra*) would be interesting.

### Multiple transmission routes

Several transmission routes have been demonstrated for brucellosis in domestic animals: horizontal direct or indirect transmission through genital secretions, aborted fetuses or products of live births (placenta, fetal fluids and vaginal exudate) from infected mothers, vertical transmission *in utero* or during birth, pseudo-vertical transmission through the consumption of colostrum or milk, and venereal transmission (Díaz-Aparicio, 2013).

Here, our data supported possible horizontal transmission through urogenital secretions, as we found positive bacterial cultures in genital swabs, genital tracts, and urine/bladders of seropositive Alpine ibex of both sexes.

We did not test the semen or epididymis of males, but positive bacterial cultures of testes observed in 12/32 (37%) males with more than 100 CFU per plate for five individuals support the ability of males to transmit the infection through venereal transmission. In seropositive naturally-infected bulls, Hill (1983) found positive bacterial cultures of testes in 2/17 (12%) of animals. Shedding in the semen is not believed to be a major mode of transmission in domestic animals (King, 1940; FAO and WHO, 1986) but spread of the infection through artificial insemination has been reported (Bendixen and Blom, 1947; Manthei et al., 1950). In the American bison (*Bison bison*), low amounts of bacteria in the semen outside the mating period did not support this transmission route, but it cannot be excluded that the amount of bacteria could increase during the mating period (Frey et al., 2013). Here, we observed high loads of *Brucella* in testicular tissues outside the mating period, so it is highly probable that it will also be the case during the rut, possibly allowing sexual transmission.

We also provided the very first indication of possible vertical transmission of *B. melitensis* in Alpine ibex, with one positive fetus out of the three fetuses analyzed. Besides, our results support a possible transmission from females to newborns through the consumption of colostrum or milk. *B. melitensis* was isolated from the udder of five females, and from 35% of supramammary lymph nodes (68% in actively infected females), whose infection is related to the presence of brucellae in milk in livestock (Philippon et al., 1971). Our results are similar to those found in naturally-infected and actively infected cattle, in which Corner et al. (1987) found 70% of positive supramammary lymph nodes. As most captures occurred during the last third of pregnancy, i.e., before the lactating period, we were not able to sample milk in the field or during necropsy. Any opportunity to sample milk in culled or captured females in the future should be seized to evaluate milk shedding.

### Shedding potential among seropositive animals

Thirty percent of the seropositive Alpine ibex we studied carried the bacteria in their urogenital organs or secretions and thus represented a potential source of bacteria for other ibex and susceptible species during the reproductive cycle at the time of capture. The proportion of active infections in urogenital organs was probably underestimated in the present study, due to the few fetuses and placentas analyzed. Moreover, our models reveal that frozen urogenital samples had a significantly lower probability of being culture-positive than fresh ones. The same trend was present but not significant in other organs. Thus, freezing carcasses before necropsy allows for a practical and efficient analysis process, but at the price of a slightly lower sensitivity of bacterial culture. qPCR could have allowed to improve the detection of the bacteria in urogenital organs, but it would not necessarily give us any further information on bacterial shedding as it could also detect DNA of dead bacteria, i.e., without any consequences on transmission.

Moreover, more than half were positive either in genital tract or in lymph nodes from the pelvic area, i.e., could be at-risk of potential shedding during future reproductive cycles. Indeed, the bacteria can persist in regional lymph nodes (Fensterbank, 1987) and be reactivated on favorable occasions (i.e., estrus, pregnancy, immunosuppression) with shedding in genital secretions and milk (European Commission, 2001).

The strong variability of bacteriological results among seropositive individuals illustrates the heterogeneity of the host-pathogen relationship, and underlines the limits of serological investigations to infer the transmission dynamics of pathogens (González-Barrio et al., 2015).

An important result of our study is that the probability of positive bacterial culture and bacterial carriage was at the highest level for the youngest animals in our sample (i.e., 2 years old) and then decreased with age, as described in wild bison (Treanor et al., 2011).

The biology of infection in ibex under 2 years of age remains unknown, as our sample did not include individuals under two because these were seldom captured and always seronegative (only five individuals under 2 captured between 2012 and 2017, and all were negative to LFIA, RBT, and CFT). However, one cannot exclude that young ibex individuals may play an unrecognized role in brucellosis dynamics since they might get infected early in life, either through transmission from infected females to viable fetus or through the colostrum or the infected environment after birth, and become shedders once sexually mature. In the future, depending on the management measures implemented to control this outbreak, analyzing young ibex might allow us to confirm that sexually immature animals may experience latent (undetected) infection, showing no or mild serological response until their sexual maturity or first gestation, as demonstrated in cattle with *B. abortus* (Plommet et al., 1973; Lapraik et al., 1975) or small ruminants with *B. melitensis* (Renoux, 1962; Grilló et al., 1997).

In females, the probability of positive bacterial culture and bacterial carriage was highest for young animals and high CFT titers. Because CFT titer is also higher in young individuals, confounding effects could be expected between age and CFT titer; however, both variables were actually selected in the final model. In domestic ruminants, it is well-known that infection with *B. melitensis* in primiparous females often leads to abortion and subsequent *Brucella* shedding during the last third of the pregnancy (Carvalho Neta et al., 2010; Godfroid et al., 2013). Besides, high shedding was associated with high CFT titers: around abortion or births, CFT titers peaked and reached high values in ewes that shed brucellae, whereas they were notably low in ewes that did not shed the bacteria (Durán-Ferrer et al., 2004). Sexual maturity in the Alpine ibex is reached at the earliest at the age of 1.5 years in females (Couturier, 1962), with first access to reproduction ranging between 1.5 and 3.5 years depending on individuals and population density (Gauthier et al., 1991). Therefore, the highest probability of bacterial shedding and the highest bacterial load in female ibex could correspond to the first pregnancy event. Most animals were captured between April and June, i.e., during the last third of pregnancy, thus in a period of potential abortion and shedding in female ibex (Gauthier et al., 1991). Our results thus suggest the probability of bacterial shedding should be at the highest in primiparous females in relation to abortion, and should decrease during subsequent pregnancies, as observed in wild bison (Treanor et al., 2011) or in domestic ruminants (Tittarelli et al., 2005). Such a scenario might explain the decreasing probability of positive bacterial culture as age increases. Unfortunately, too few pregnant females were integrated in our sample to properly test a relationship between pregnancy status and *Brucella* shedding. Additionally, shedding in old females could have been underestimated, because too few captures occurred during the birth period, when shedding occurs in vaginal discharges and birth products. In contrast, evaluation of shedding in young females was probably more accurate, as captures occurred during the abortion period.

Interestingly, the probability of bacterial shedding and the bacterial load did not vary with CFT titers in males, i.e., males with low CFT titers could carry high amounts of *Brucella*. However, here most captures occurred in April-June. The behavior of *Brucella* shedding in sexually active males during the mating period (end of November to January) remains unknown. Active shedding with high titers of antibodies might happen then, in a similar way as females around abortion or birth. Season of sampling (April-June vs. October-November) could also have influenced the bacterial load; unfortunately, too few animals were sampled in autumn to allow us to explore the effect of season on our results.

Males generally had higher probability of positive culture and higher bacterial load than females (Figure 3). However, it is unlikely that males play a more important role than females, as this result needs to be put in perspective with the possible transmission routes. Indeed, males, even with high bacterial loads, could not be responsible for disease transmission outside the mating period, as their only possible route of transmission is venereal transmission, each contact infecting a single female. In contrast, females can contaminate many susceptible individuals through indirect transmission following abortion or births.

Finally, we failed to detect any change in this shedding pattern before and after a mass culling had been performed in this ibex population, thus the selective captures performed before 2013 did not result in any obvious change in the bacterial shedding pattern.

### Two different epidemiological roles for males and females?

Our results on shedding and previous results on the spatial behavior and contact patterns of Alpine ibex in the study population (Marchand et al., 2017) support two different epidemiological roles for males and females. We argue that on the one hand, females play a role of maintenance of the infection inside each spatial unit of the Bargy Massif, by shedding bacteria especially through abortion during the first gestation following infection and then through parturition following subsequent gestations. On the other hand, males of all ages tend to visit all subunits during the mating period (Marchand et al., 2017) and thus could play a role of transmission between spatial units through venereal transmission. This kind of pattern was hypothesized for instance for Aujeszky's disease in wild boar, with venereal male-to-female transmission during mating and oro-nasal female-to-female transmission only outside mating (Ruiz-Fons et al., 2007; González-Barrio et al., 2015). This difference could be even more marked in our case, as the efficacy of female-to-female transmission through indirect transmission following abortion or birth is likely to be high, consequently playing a crucial role in maintaining the infection inside spatial units.

### Management options

As highlighted throughout the discussion, the biology of infection remains unknown as regards young or seronegative ibex for instance. Besides, we used here a cross-sectional study, as it is not easily conceivable to use experimental infection for the study of brucellosis in Alpine ibex (for instance, it would require building BSL-3 facilities respecting the welfare of a wild caprine, which in addition is a protected species in France). Therefore, further studies are needed to better understand the infection dynamics in the whole population. However, our results provide a new insight in the infection epidemiology, which is critical for future development of control strategies. Indeed, heterogeneity due to age and sex can impact the efficacy of management methods such as culling based on the serological status of individuals or groups, for example by skewing population age structure in favor of highly susceptible juveniles (Bolzoni et al., 2007). In the present case, removing old animals might have contributed to limited efficacy of the mass culling performed in 2013 (focused on animals aged more than 5 years old) since older animals were not necessarily the ones shedding the most bacteria. In the Yellowstone area, test-and-cull strategies were indeed most successful at reducing seroprevalences in bison when targeting young seropositive females (Ebinger et al., 2011). Modeling approaches, including the different kinds of data (on Brucella epidemiology and ibex demography) that have been collected in the field from 2012 onwards, will now be used to infer disease management principles that may be relevant in this and other cases.

## Author contributions

BG-B, YG, JH, SR, CP, and MJ designed the study. PF, EP, and CT collected the data during the field work, and YG, GR, and M-NB performed laboratory analyses. SL and PF filtered, entered and prepared the data for the analysis. SL, PF, and EG-F performed the analysis. SL, PF, SR, AT, EG-F, and CT interpreted the data. SL wrote the manuscript. SR and JH supervised the monitoring program conducted by the French Hunting and Wildlife Agency. All authors participated in drafting the manuscript or revising it critically for important intellectual content. All the authors approved the submitted version of the manuscript.

### Conflict of interest statement

The authors declare that the research was conducted in the absence of any commercial or financial relationships that could be construed as a potential conflict of interest.
